# The subcellular topology of the RNAi machinery is multifaceted and reveals adherens junctions as an epithelial hub

**DOI:** 10.21203/rs.3.rs-5837046/v1

**Published:** 2025-02-21

**Authors:** Joyce Nair-Menon, Christina Kingsley, Houda Mesnaoui, Peter Lin, Kyrie Wilson, Bärbel Rohrer, Antonis Kourtidis

**Affiliations:** Medical University of South Carolina (MUSC); Medical University of South Carolina (MUSC); Medical University of South Carolina (MUSC); Medical University of South Carolina (MUSC); Medical University of South Carolina (MUSC); Medical University of South Carolina (MUSC); Medical University of South Carolina (MUSC)

## Abstract

The RNA interference (RNAi) machinery is a key cellular mechanism catalyzing biogenesis and function of miRNAs to post-transcriptionally regulate mRNA expression. The RNAi machinery includes a set of protein complexes with subcellular localization traditionally presented in a uniform fashion: the microprocessor processes miRNAs in the nucleus, whereas the DICER and the RNA-induced silencing complex (RISC) further process and enable activity of miRNAs in the cytoplasm. However, several studies have identified subcellular patterns of RNAi components that deviate from this model. We have particularly shown that RNAi complexes associate with the adherens junctions of well-differentiated epithelial cells, through the E-cadherin partner PLEKHA7. To assess the extent of these subcellular topological patterns, we examined subcellular localization of the microprocessor and RISC in a series of human cell lines and normal human tissues. Our results show that junctional localization of RNAi components is a broad characteristic of well-differentiated epithelia, but it is absent in transformed or mesenchymal cells and tissues. We also find extensive localization of the microprocessor in the cytoplasm, as well as of RISC in the nucleus. These findings expose a RNAi machinery with multifaceted subcellular topology that may inform its physiological role and calls for updating of the current models.

## Introduction

The RNA interference (RNAi) machinery is a conserved mechanism, responsible for the biogenesis and function of a class of short RNAs, called microRNAs (miRNAs)^1–3^. miRNAs bind to mRNAs through base pair complementarity resulting in mRNA translational suppression or degradation, in this way adjusting the flow of genetic information from DNA to protein in a post-transcriptional manner^4^. Since the discovery of RNAi in the late 1990s^5^, a substantial body of work has described in exquisite detail a set of RNA-binding complexes as the key components of the RNAi machinery. These complexes are: a) the microprocessor complex, which mainly consists of two protein components, DROSHA and DGCR8; b) the DICER complex; and c) the RNA-induced silencing complex (RISC), which includes AGO2, the main RNAi catalytic component mediating the targeting of mRNAs by miRNAs, as well as its key functional partner GW182^1–3,6,7^. The microprocessor is responsible for mediating the first step in miRNA biogenesis by catalyzing processing of primary miRNAs (pri-miRNAs) to precursor miRNAs (pre-miRNAs), which are then further processed by DICER to generate mature double-stranded miRNAs. One of the two strands of the miRNA duplex is eventually loaded to RISC, which uses it to target a mRNA, effectively blocking it from being translated to a protein, or eventually resulting in its degradation^1–3^.

An aspect of the RNAi biology that has gained significant attention as being critical for the function of RNAi complexes, is their subcellular localization. This localization was examined in a number of early studies and has since appeared in a uniform format in the bibliography: the microprocessor complex acts in the cell nucleus to process pri-miRNAs, which are then exported in the cytoplasm to be further processed by DICER, and eventually become engaged by RISC, again in the cytoplasm^1–4,8–11^. More specified subcellular compartments that these complexes act within have also shown to be: a) the paraspeckles and Cajal bodies in the nucleus, regarding the microprocessor complex^12,13^; and b) p-bodies and stress granules for the RISC complex and AGO2 in the cytoplasm^14–16^. However, a significant number of studies have also challenged this view, showing that: a) DROSHA and the microprocessor also localize to the cytoplasm, e.g. as a response to viral infection and a defense mechanism, or due to the presence of functional, alternative splicing – derived variants^17–21^; b) AGO2 and RISC can also localize to the nucleus, where they silence mRNAs in embryonic stem cells, they can suppress transposons in quiescent splenocytes, regulate transcriptional gene silencing upon senescence in fibroblasts, or during viral infection, and bind to chromatin in germline cells to regulate gene expression^22–27^. In fact, presence and function of AGO2 and of RISC in the cell nucleus was identified and discussed early on in the field^2,11,28^.

Along these lines, we have also revealed localization of the microprocessor, DICER, and RISC at adherens junctions of well-differentiated epithelial cells. We particularly showed that the adherens junction - associated RNAi complexes engage certain sets of miRNAs, to mediate localized miRNA processing and miRNA-mediated silencing of a set of pro-tumorigenic mRNAs, in this way maintaining the well-differentiated epithelial phenotype^29–31^. Adherens junctions are specialized cell-cell adhesion structures that form at apical areas of cell-cell contact^32,33^. The core adherens junction component is typically a member of the family of classical cadherins, such as E-cadherin, the predominant cadherin family member in epithelial tissues^32,33^. Cadherins are transmembrane proteins that are fundamental to establish cell-cell adhesion. Intracellularly, cadherins recruit members of the catenin protein family, namely p120 catenin (p120), β-catenin, and α-catenin, the latter of which tethers the whole structure to a tensile circumferential actomyosin cytoskeletal ring^32,33^. In this way, adherens junctions are critical for tissue formation, architecture, and integrity^32,33^. We have shown that recruitment and functionality of key RNAi components at adherens junctions depends on the p120 binding partner PLEKHA7, which supports adherens junction integrity, as well as on extracellular matrix influences and on actomyosin integrity, tethering RNAi regulation to the architectural and mechanosensitive role of adherens junctions^29,34,35^.

Altogether, these findings reveal association of RNAi complexes with adherens junctions and subcellular compartments other than the ones originally described in the bibliography and demonstrate that subcellular localization of RNAi complexes can enable and inform their function. However, a critical question emerging from these findings is the extent to which these subcellular localization patterns of RNAi complexes exist and whether these are cell- or tissue-specific, possibly explaining the diverse observations between different studies. For example, only a limited set of cell lines, such as HEK293, HeLa, or fibroblasts, has been traditionally used in most of the studies where subcellular localization of these complexes has been assessed^8–11^. To shed more light into this conundrum, we set off to examine subcellular localization of the microprocessor and RISC complexes, particular focusing on the junctional localization of these complexes, in a series of well-differentiated and transformed cell lines, including some that are most commonly used in the fields or RNAi or epithelial and endothelial biology, as well as to a set of tissues.

## Results

### Rationale for the experimental models used

To examine subcellular localization of RNAi components and to specifically interrogate prevalence of their localization at adherens junctions, we collected a series of cell lines that have either been historically used to study adherens junctions, or RNAi function, or both. This set of cell lines included: a) well-differentiated epithelial cell lines that are commonly used in the field of cell-cell adhesion, including well-differentiated colon epithelial Caco2 cells, breast immortalized MCF10A cells, retinal pigment epithelial (ARPE-19) cells, primary Human Umbilical Vein Endothelial (HUVEC) cells, and the spontaneously immortalized human epidermal keratinocyte HaCaT cell line; b) cell lines that have been used as models to study RNAi, such as liver cancer HepG2, cervical cancer HeLa cells, and osteosarcoma U2OS cells; and c) human intestinal smooth muscle (HSIM) cells, as a fibroblast model. Caco2 cells are derived from a primary adenocarcinoma of the colon, however, they fully differentiate in culture and are excellent models of the well-differentiated epithelium traditionally used to study cell-cell junctions^36–38^. We have made key observations on the adherens junction – associated RNAi complexes using these cells, as well as in normal human colon tissues, therefore, we used these colon cells and tissues as our reference for the adherens junction – related RNAi observations^29–31,34,35^. We have also previously identified junctional localization of RNAi complexes using the Madin-Darby canine kidney (MDCK) cells^29,30^, another excellent model of the well-differentiated epithelium, also frequently used in the field of cell-cell adhesion. However, these cells were not used in this study, since we have already published our observations on junctional RNAi localization in these cells and to put our focus specifically on human cell lines and tissues. In all cases, we cultured all cell lines until they were fully confluent, before performing immunofluorescence, to allow them to form cell-cell contacts and adherens junctions, which allowed us to compare junctional RNAi localization among all of them. We have previously extensively verified subcellular and specifically junctional localization patterns of RNAi components in colon and MDCK cells, by using a combination of multiple antibodies, knockdown studies, and ectopically expressing constructs^29–31,34,35^. Based on the above, we here moved on to assess subcellular localization of the key RNAi components DROSHA, DGCR8, AGO2, GW182, as well as of PLEKHA7, their protein anchor to adherens junctions, across the different cell lines and tissues that we collected.

### PLEKHA7 localizes at adherens junctions specifically in well-differentiated cells

We have previously found that recruitment of RNAi complexes to the adherens junctions is mediated by the p120 binding partner PLEKHA7 and that depletion of this protein results in loss of junctional localization and decreased functionality of RNAi components^29,30,34^. We have also shown that PLEKHA7 mislocalization or absence in transformed colon cancer cell lines and tumors also correlates with absence of junctional localization of RNAi complexes and that PLEKHA7’s re-expression in aggressive colon cancer cells can restore it^31^. Overall, PLEKHA7 is responsible for stabilizing adherens junctions, downstream of the cadherin-catenin complex, through stabilizing the barrier function and the actomyosin cytoskeleton^29,34^. Therefore, to obtain insights into the overall status of adherens junctions in these cell lines, and to be able to further associate PLEKHA7’s status with RNAi recruitment to the adherens junctions, we first examined subcellular localization of this protein in the set of cell lines that we have collected, through using immunofluorescence and confocal microscopy. To be able to assess junctional localization in these stainings, as well as throughout all the stainings involving RNAi components, we co-stained cells with the core junctional marker p120. Indeed, p120 or E-cadherin localization to the junctions is not substantially affected by PLEKHA7 mislocalization or depletion^29,30^. We did not use E-cadherin as a junctional marker here, since these cell lines come from different tissues (colon, endothelial, breast, skin) or are transformed, and therefore express different members of the cadherin family. For example, Caco2 cells express E-cadherin, the epithelial-specific cadherin, whereas HUVECs express VE-cadherin, the endothelial-specific one. However, p120 associates with all classical cadherins and therefore can be used as a universal junctional marker across the different cell types examined here^39^.

**Figure 1:**
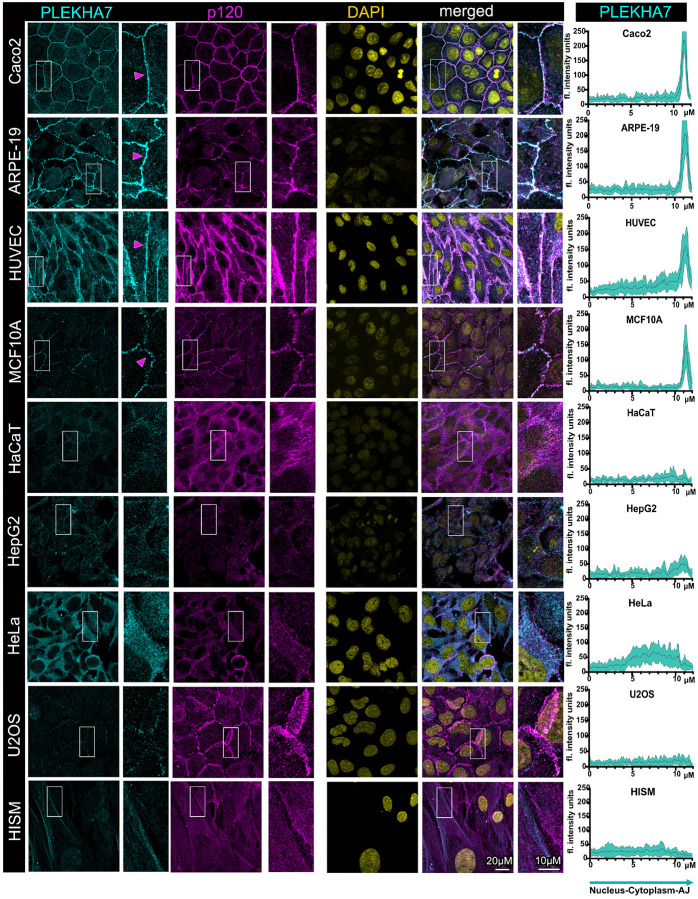
Junctional localization of the adherens junction component and RNAi-interacting partner PLEKHA7 is present in well-differentiated cells but absent in transformed cells and fibroblasts. Immunofluorescence and confocal microscopy of well-differentiated colon epithelial Caco2 cells, retinal epithelial ARPE-19 cells, human endothelial HUVEC cells, breast immortalized epithelial MCF10A cells, immortalized HaCaT keratinocytes, HepG2 liver cancer cells, cervical cancer HeLa cells, osteosarcoma U2OS cells, as well as intestinal HISM fibroblasts, stained for PLEKHA7 and p120; DAPI is the nuclear stain. Merged images are shown on the right. Enlarged image insets are denoted with white boxes and shown to the right of their respected images. Magenta arrowheads indicate junctional localization. Scale bars are shown at the bottom right image and inset and are the same for all images and insets. Line scan quantifications spanning the nuclear, cytoplasmic, and junctional compartments per cell line are shown on the right column; the black line represents the average fluorescence intensity of 15 cells from three fields for each cell line, whereas the cyan area represents the standard deviation.

Similarly to our past observations, our examination here confirmed the predominant and almost exclusive localization of PLEKHA7 at adherens junctions in fully confluent Caco2 cells (Fig. 1). These cells form overall linear, mature, and tensile adherens junctions when fully differentiated. We obtained similar results when we co-stained PLEKHA7 and p120 in the retinal pigment epithelial ARPE-19 cells, in the endothelial HUVEC cells, and in the breast epithelial MCF10A cells (Fig. 1). All these cell lines are also well-differentiated and form well-organized adherens junctions, as denoted by the strong junctional staining of p120 (Fig. 1). Notably, PLEKHA7 exhibits some degree of cytoplasmic localization in HUVEC cells, although it is almost exclusively junctional in ARPE-19 and MCF10A cells (Fig. 1). However, localization of PLEKHA7 to the junctions was entirely absent in the HaCaT keratinocytes, although these cells are also considered well-differentiated (Fig. 1). Examination of PLEKHA7’s total protein levels by western blot showed that they are overall lower in HaCaT cells, compared to Caco2 or ARPE-19 cells (Fig. S1). Similarly, PLEKHA7’s total levels also seem to be downregulated and its junctional localization is absent from in HepG2, HeLa, U2OS, and HISM cells (Fig. 1). In all these cases, including in the well-differentiated HaCaT cells, p120 still localizes at areas of cell-cell contact, however, it is more diffused, denoting lack of adherens junction maturation. p120 total protein levels are overall more uniform among these cell lines, with some lower levels observed in HaCaT and HepG2 cells (Fig. S1). However, there seem to be notable differences in the respective p120 isoforms expressed in these cells, with Caco2, ARPE-19, and MCF10A cells primarily expressing isoform 3, which is associated with more stable adherens junctions, whereas HeLa and U2OS cells seem to primarily express isoform 1, which is associated with less stable junctions and pro-tumorigenic phenotypes^39^ (Fig. S1). In summary, these results show that junctional localization of PLEKHA7 is strongly observed in the well-differentiated Caco2, ARPE-19, HUVEC, and MCF10A cells, whereas it is absent from the other cell lines, surprisingly including the also well-differentiated HaCaT cells, in all latter cases correlating with poor junction maturation.

### The microprocessor exhibits distinct junctional and subcellular localization patterns in different cell types

**Figure 2:**
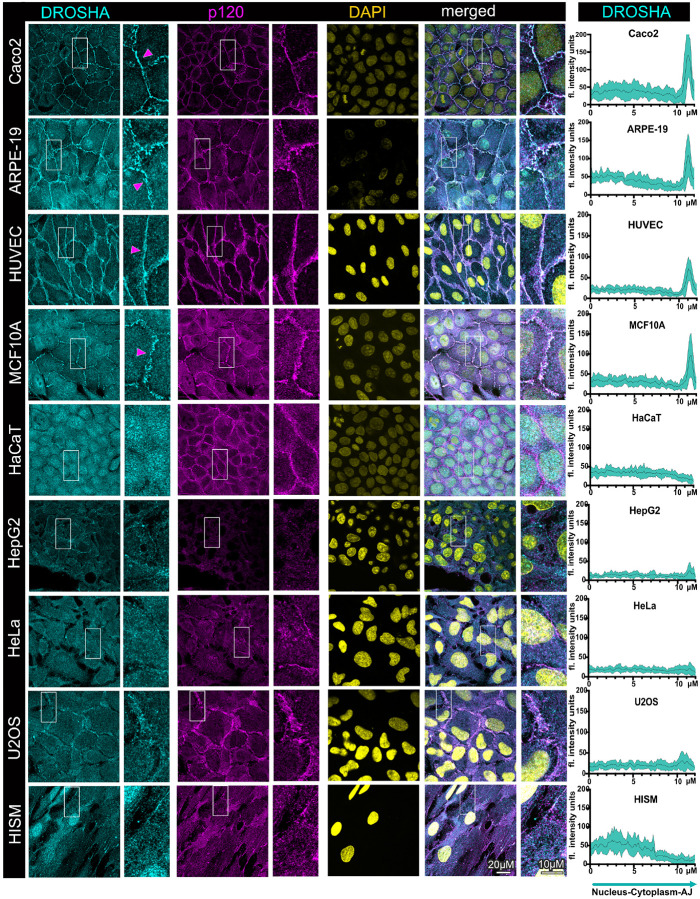
The key microprocessor enzyme DROSHA exhibits junctional localization in well-differentiated cells, but only cytoplasmic or nuclear localization in transformed cells and fibroblasts. Immunofluorescence and confocal microscopy of well-differentiated colon epithelial Caco2 cells, retinal epithelial ARPE-19 cells, human endothelial HUVEC cells, breast immortalized epithelial MCF10A cells, immortalized HaCaT keratinocytes, HepG2 liver cancer cells, cervical cancer HeLa cells, osteosarcoma U2OS cells, as well as intestinal HISM fibroblasts, stained for DROSHA and p120; DAPI is the nuclear stain. Merged images are shown on the right. Enlarged image insets are denoted with white boxes and shown to the right of their respected images. Magenta arrowheads indicate junctional localization. Scale bars are shown at the bottom right image and inset and are the same for all images and insets. Line scan quantifications spanning the nuclear, cytoplasmic, and junctional compartments per cell line are shown on the right column; the black line represents the average fluorescence intensity of 15 cells from three fields for each cell line, whereas the cyan area represents the standard deviation.

**Figure 3:**
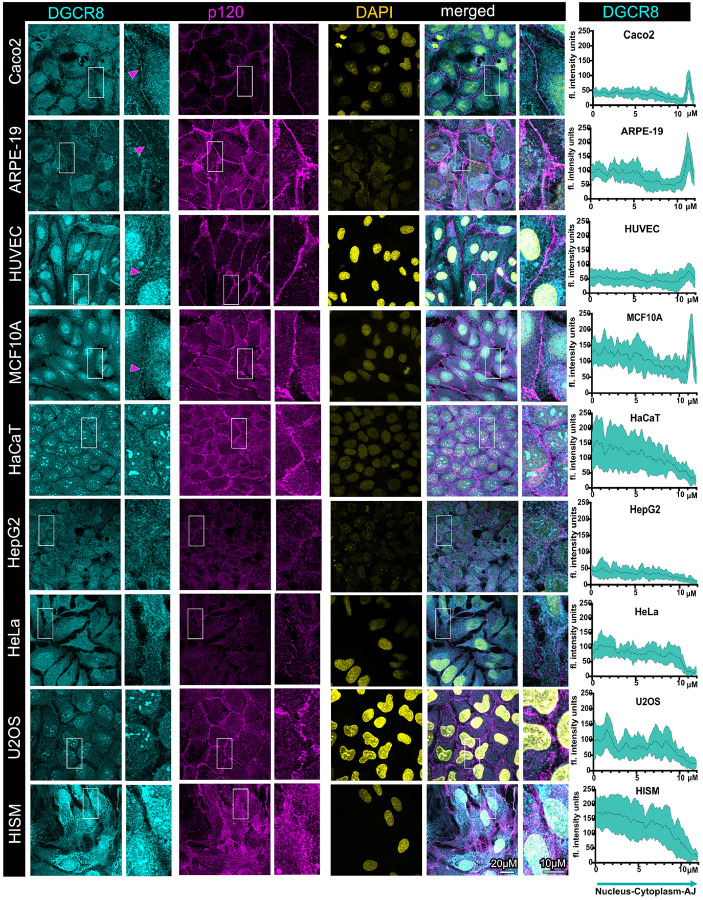
The core microprocessor component DGCR8 exhibits junctional localization in well-differentiated cells, but only cytoplasmic or nuclear localization in transformed cells and fibroblasts. Immunofluorescence and confocal microscopy of well-differentiated colon epithelial Caco2 cells, retinal epithelial ARPE-19 cells, human endothelial HUVEC cells, breast immortalized epithelial MCF10A cells, immortalized HaCaT keratinocytes, HepG2 liver cancer cells, cervical cancer HeLa cells, osteosarcoma U2OS cells, as well as intestinal HISM fibroblasts, stained for DGCR8 and p120; DAPI is the nuclear stain. Merged images are shown on the right. Enlarged image insets are denoted with white boxes and shown to the right of their respected images. Magenta arrowheads indicate junctional localization. Scale bars are shown at the bottom right image and inset and are the same for all images and insets. Line scan quantifications spanning the nuclear, cytoplasmic, and junctional compartments per cell line are shown on the right column; the black line represents the average fluorescence intensity of 15 cells from three fields for each cell line, whereas the cyan area represents the standard deviation.

We then examined localization of the microprocessor in the same set of cell lines. The microprocessor is comprised of two main components, DROSHA and DGCR8. As we have shown before, localization of these proteins to cell-cell junctions is evident in confluent, well-differentiated Caco2 cells, which we confirmed in this study (Figs. 2, 3)^29,31^. In these cells, we also observed that there is a substantial cytoplasmic localization of these proteins, and only a partially nuclear one, which is the localization that is almost exclusively mentioned in the literature for the microprocessor (Figs. 2, 3). Similarly, we also observed strong junctional localization of DROSHA and DGCR8 in the well-differentiated ARPE-19, HUVEC, and MCF10A cells, with the junctional localization of DROSHA being more pronounced in all cases, compared to that of DGCR8 (Figs. 2, 3). The nuclear localization of the microprocessor is more apparent in the ARPE-19 and MCF10A cells, where it is particularly manifested by the strong nuclear localization of DGCR8 (Figs. 2, 3). Notably, junctional localization of DROSHA and DGCR8 is somewhat weaker in HUVEC cells (Figs. 2, 3). These junctional localization patterns correlate with that of PLEKHA7 in the same cell lines, where PLEKHA7 is indeed more diffused and not solely junctional in HUVEC cells (Fig. 1). These data demonstrate that the microprocessor exhibits significant junctional localization in these well-differentiated cells, and only partially nuclear, substantially deviating from the predominant notion regarding the subcellular localization of the microprocessor.

In contrast, junctional localization of DROSHA and DGCR8 is entirely absent in HaCaT, HepG2, HeLa, U2OS, and HISM cells (Figs. 2, 3). The nuclear localization of the microprocessor is more apparent in HaCaT, U2OS, and HISM cells, again especially regarding DGCR8, whereas the predominant localization in HepG2 and HeLa cells for both DROSHA and DGCR8 is cytoplasmic (Figs. 2, 3). Interestingly, DGCR8 and to a lesser extend DROSHA, exhibit strong localization in nuclear speckles in MCF10A, HaCaT, and in U2OS cells (Figs. 2, 3). DROSHA also exhibits a more obvious speckle-like pattern in the HaCaT nuclei, however, this is somewhat masked by the broader and strong DROSHA abundance in the nucleoplasm (Figs. 2, 3). Localization of the microprocessor to nuclear condensates, such as Cajal bodies and paraspeckles, has been reported^12,13^. Protein examination by western blot also showed that HepG2 cells seem to express alternative isoforms of DROSHA, whereas ARPE-19, HeLa, U2OS, and MCF10A cells express alternative isoforms of DGCR8. However, these alternative isoforms do not seem to correlate with their different localization patterns in these cells. In summary, the data show that the microprocessor exhibits strong junctional localization in the well-differentiated cell lines, with the exception of HaCaT cells, correlating with PLEKHA7’s junctional localization in the same cell lines. The data also show that DROSHA and DGCR8 are primarily cytoplasmic or nuclear in the transformed or the fibroblast cell lines. There are also some notable differences in the subcellular patterns of DROSHA and DGCR8, with DROSHA exhibiting stronger junctional localization overall, whereas DGCR8 relatively stronger localization in the nucleus and particularly in nuclear speckles.

### The RISC is junctional with localization patterns both similar and distinct to PLEKHA7 and the microprocessor

**Figure 4:**
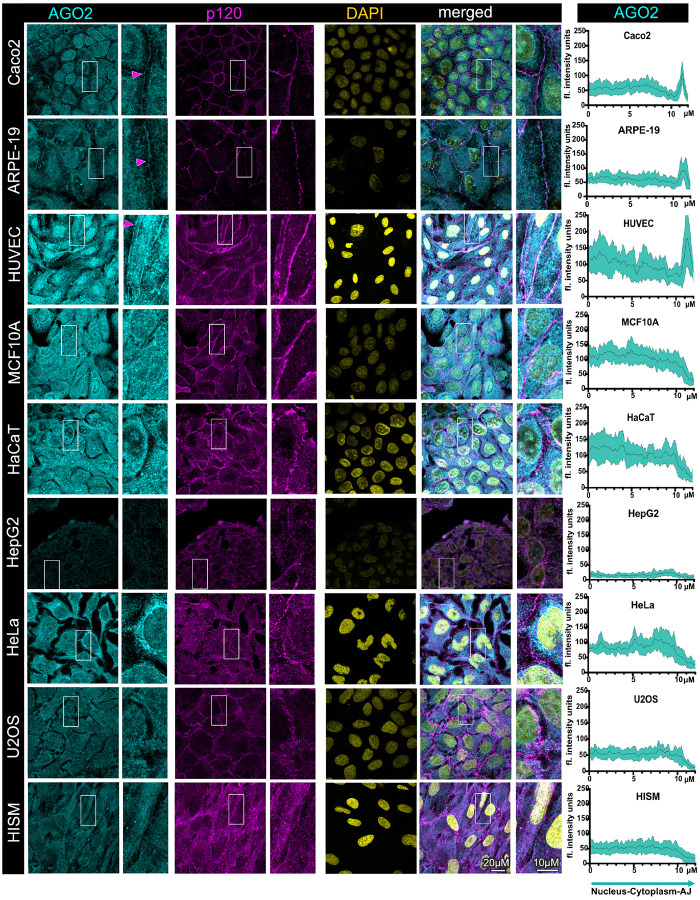
AGO2, the core RISC catalytic component exhibits junctional localization in the subset of well-differentiated cell lines that form mature adherens junctions, but it is cytoplasmic or partially nuclear in all other cell lines. Immunofluorescence and confocal microscopy of well-differentiated colon epithelial Caco2 cells, retinal epithelial ARPE-19 cells, human endothelial HUVEC cells, breast immortalized epithelial MCF10A cells, immortalized HaCaT keratinocytes, HepG2 liver cancer cells, cervical cancer HeLa cells, osteosarcoma U2OS cells, as well as intestinal HISM fibroblasts, stained for AGO2 and p120; DAPI is the nuclear stain. Merged images are shown on the right. Enlarged image insets are denoted with white boxes and shown to the right of their respected images. Magenta arrowheads indicate junctional localization. Scale bars are shown at the bottom right image and inset and are the same for all images and insets. Line scan quantifications spanning the nuclear, cytoplasmic, and junctional compartments per cell line are shown on the right column; the black line represents the average fluorescence intensity of 15 cells from three fields for each cell line, whereas the cyan area represents the standard deviation.

**Figure 5:**
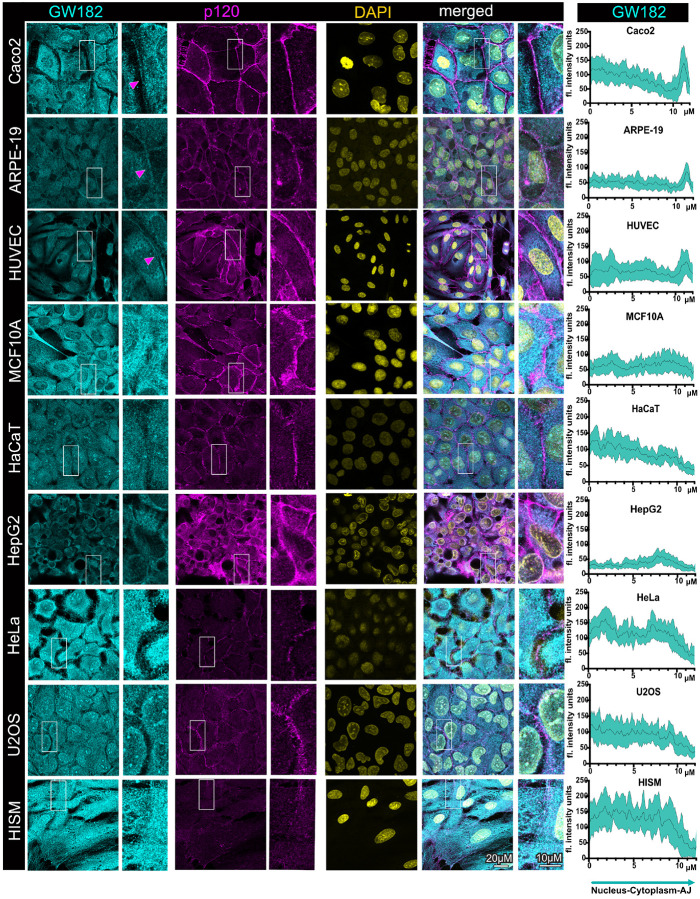
The key RISC component GW182 exhibits junctional localization in the subset of well-differentiated cell lines that form mature adherens junctions, but it is cytoplasmic or partially nuclear in all other cell lines. Immunofluorescence and confocal microscopy of well-differentiated colon epithelial Caco2 cells, retinal epithelial ARPE-19 cells, human endothelial HUVEC cells, breast immortalized epithelial MCF10A cells, immortalized HaCaT keratinocytes, HepG2 liver cancer cells, cervical cancer HeLa cells, osteosarcoma U2OS cells, as well as intestinal HISM fibroblasts, stained for GW182 and p120; DAPI is the nuclear stain. Merged images are shown on the right. Enlarged image insets are denoted with white boxes and shown to the right of their respected images. Magenta arrowheads indicate junctional localization. Scale bars are shown at the bottom right image and inset and are the same for all images and insets. Line scan quantifications spanning the nuclear, cytoplasmic, and junctional compartments per cell line are shown on the right column; the black line represents the average fluorescence intensity of 15 cells from three fields for each cell line, whereas the cyan area represents the standard deviation.

Next, we interrogated the subcellular localization of the two core components of RISC, namely AGO2 and GW182. AGO2 is the key RNAi enzymatic component catalyzing miRNA-mediated mRNA silencing, whereas GW182 is critical for AGO2’s silencing function^6,7^. This complex and its components have been previously described as primarily cytoplasmic^1–4,10,11^. However, as with the microprocessor, we observe junctional localization of both AGO2 and GW182 in the well-differentiated Caco2, APRE-19, and HUVEC cells, in addition to their cytoplasmic distribution (Figs. 4, 5). These findings are in agreement with our previous observations regarding junctional recruitment of the RISC in the well-differentiated Caco2 cells^30,31,34^ and correlate with PLEKHA7’s junctional localization in the Caco2, APRE-19, and HUVEC cell lines (Fig. 1). However and surprisingly, MCF10A cells do not exhibit junctional localization of either AGO2 or GW182 (Figs. 4, 5), although they are well-differentiated, and despite exhibiting junctional localization of PLEKHA7 (Fig. 1), which we have shown that is required to recruit AGO2 to the junctions^30,34^. HaCaT cells also do not exhibit any junctional localization of the RISC components, similarly to the microprocessor, and despite these cells also being well-differentiated (Figs. 4, 5). HepG2, HeLa, U2OS, and HISM cells also lack junctional localization of AGO2 and GW182; in all these cell lines, localization of AGO2 and GW182 is predominantly cytoplasmic (Figs. 4, 5). Intriguingly, it seems that both AGO2 and GW182 are also nuclear in HUVEC, MCF10A, HaCaT, U2OS, and HeLa cells, where they exhibit a speckle-like nuclear localization pattern, similarly to DROSHA and DGCR8 (Figs. 4, 5). The nuclear localization of AGO2 has been previously reported^22–27^, particularly in HeLa and HaCaT cells^40^, but not in this specialized, speckle-like pattern. Western blot analysis didn’t show any AGO2 isoforms or different total expression levels, although GW182 seems to have an additional isoform in HepG2 and HISM cells (Fig. S1). Taken together, the findings demonstrate that the core RISC components AGO2 and GW182 exhibit distinct localization patterns in the in the well-differentiated cells, where they are junctional in Caco2, ARPE-19, and HUVEC cells, but not in MCF10A or HaCaT cells. Furthermore, similarly to PLEKHA7 and the microprocessor, they are entirely absent from cell-cell junctions of the transformed or mesenchymal cells that we examined. Finally, AGO2 and GW182 also substantially localize in the nucleus and in specific speckle-like patterns, exhibiting an overall subcellular distribution that is much broader than the one predominantly reported.

### Junctional localization of RNAi components is widespread and primarily observed at apical areas of human pithelial tissues

Next, we sought to examine localization of RNAi components in normal human tissues. We particularly focused our examination in organs with epithelial surfaces, since our cell line examination showed that RNAi components are junctional primarily in well-differentiated epithelial cells. For this reason, we collected tissues from the colon, liver, pancreas, kidney, bladder, lung, and esophagus. These tissues include extensive epithelial compartments that are representative of columnar (colon), cuboidal (liver, pancreas, kidney), transitional (bladder), squamous (lung), and stratified (esophagus) epithelium. NCBI expression data confirm that PLEKHA7 and these RNAi markers are all expressed in these tissues, with seemingly lower levels in the liver and pancreas (Fig. S2). We then developed a multiplex immunofluorescence assay to be able to simultaneously assess co-localization of our markers of interest on the same tissue, and to be able to also include a nuclear marker (DAPI). We optimized this protocol to simultaneously stain for: a) DROSHA and AGO2, which are the key microprocessor and RISC components, respectively; b) PLEKHA7, the adherens junction component associated with the RNAi machinery; and c) E-cadherin, as the epithelial-specific core adherens junction component, to highlight the epithelial areas of the tissues under examination. Although in the past we have successfully stained for these markers in colon tissue and tumors that were fresh frozen^31^, here, we optimized this protocol particularly for formalin-fixed, paraffin-embedded tissues (FFPE), where tissue structure is better maintained.

**Figure 6:**
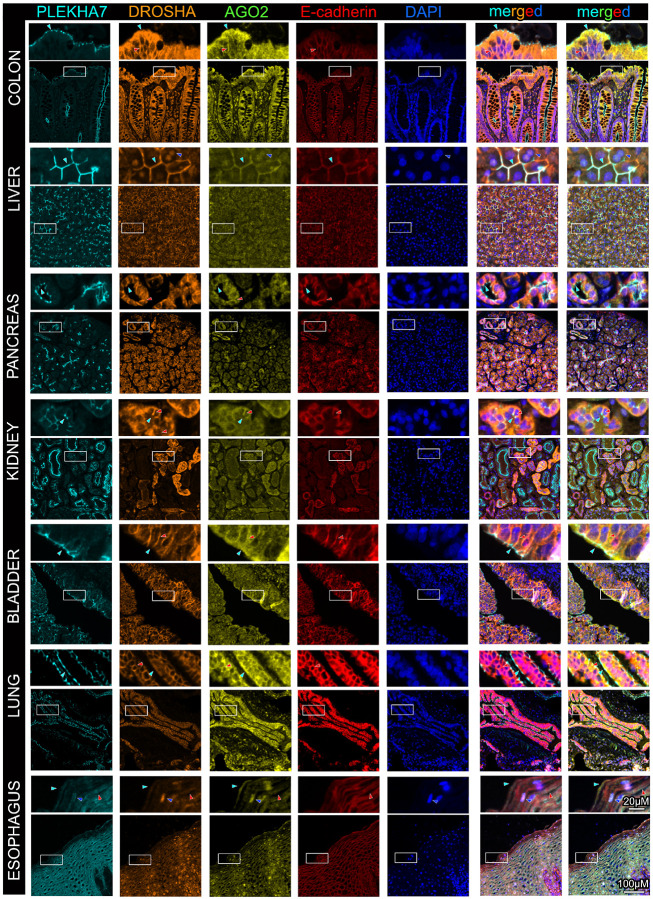
RNAi components are enriched at areas of cell-cell contact in well-differentiated human epithelial tissues. Multiplex immunofluorescence staining for the junctional markers PLEKHA7 and E-cadherin, and for the key RNAi components DROSHA and AGO2, in normal human tissues from the colon, liver, pancreas, kidney, bladder, lung, esophagus. DAPI is the nuclear stain. Enlarged image insets are denoted with white boxes and shown on top of their respected images. Light blue arrowheads indicate apical junctional staining; red arrowheads indicate lateral junctional staining; deep blue arrowheads indicate nuclear staining. Scale bars are shown at the bottom right image and inset and are the same for all images and insets.

We first examined human colon tissue, since we have previously seen robust apical junctional localization of PLEKHA7 and of RNAi markers in these tissues, as well as in the polarized monolayers of well-differentiated colon epithelial Caco2 cells^29–31^. Our analysis showed that in the colon, PLEKHA7 appears in puncta that are specifically localized only in the epithelium and at the very apical areas of the crypts at the brush border, where the well-differentiated cells reside, forming the barrier (Fig. 6). In contrast, E-cadherin also localizes at lateral areas of cell-cell contact in the epithelial crypts (Fig. 6), as expected and as we have previously reported^31^. Interestingly, although both DROSHA and AGO2 are expressed throughout the epithelium, as well as in the stroma, they are strongly enriched at apical areas of the colonic epithelium at areas of cell-cell contact, similarly to PLEKHA7 (Fig. 6). However, their distribution is not limited to the very apical puncta where PLEKHA7 localizes but is further extended at more lateral areas of cell-cell contact in the crypts, partially overlapping with E-cadherin, especially regarding DROSHA (Fig. 6). In contrast, both DROSHA and AGO2 exhibit a solely cytoplasmic and occasionally nuclear localization in the lamina propria of the colonic tissue (Fig. 6). In the liver, we observed strong localization of DROSHA and AGO2 at adherens junctions of epithelial cells, marked by the strong overlap both with E-cadherin and PLEKHA7, although PLEKHA7 again exhibits a more specific polarized pattern (Fig. 6). Interestingly, primarily DROSHA and to some extend AGO2, also exhibit nuclear localization in these cells (Fig. 6). Distribution of DROSHA and AGO2 was also junctional in the pancreatic acini and the kidney tubules, overlapping both with the more apically localized PLEKHA7 and the laterally present E-cadherin (Fig. 6). In the bladder, DROSHA is junctional and strongly expressed in the epithelium, largely co-localizing with the E-cadherin, whereas AGO2’s junctional localization is more apical, overlapping with PLEKHA7 (Fig. 6). AGO2 is also significantly expressed in the bladder stroma, where it is exclusively cytoplasmic (Fig. 6). In the lung epithelium, AGO2 is strongly apical, overlapping with PLEKHA7, and occasionally found at lateral adherens junctions, overlapping with E-cadherin (Fig. 6). DROSHA exhibits primarily lateral junctional localization in the lung epithelium, as indicated again by E-cadherin co-localization (Fig. 6). In contrast, DROSHA and AGO2 are cytoplasmic or nuclear in the lung stroma (Fig. 6). Finally, AGO2 and, to some extent, DROSHA, localize at the apical most areas of cell-cell contacts of the esophageal stratified epithelium, together with PLEKHA7, whereas they again become cytoplasmic and nuclear at the more basal layers of the epithelium (Fig. 6). Contrary to the other epithelial tissues, PLEKHA7 is abundantly expressed in the more basal areas of the stratified esophageal epithelium, where it is entirely cytoplasmic (Fig. 6).

Taken together, examination of these tissues revealed that junctional localization of DROSHA and AGO2 is widespread in epithelia, particularly at the most apical areas of these tissues, occasionally extending at lateral areas of cell-cell contact. Indeed, there is some variability in the apical vs lateral junctional localization from tissue to tissue regarding DROSHA and AGO2. Still, and taken together with the cell line findings, junctional localization of RNAi components seems to be a hallmark of well-differentiated epithelia, whereas cytoplasmic or nuclear DROSHA and AGO2 are the only modes of localization present in the stromal, mesenchymal cells and tissues.

## Discussion

Our findings from the cell line and the tissue examination reveal subcellular RNAi localization patterns that are substantially more variable than presented in the traditional model^1–3^. Firstly, we found that junctional localization of RNAi components is a widespread feature of well-differentiated epithelia, whereas this localization is entirely absent from mesenchymal cells and tissues. In these latter tissues, the main modes of localization of RNAi complexes are the cytoplasmic or nuclear, which have been previously reported to some extent for both the microprocessor and the RISC^8–11,17–27^. Still, our observations reveal a more widespread presence of the microprocessor in the cytoplasm and of the RISC in the nucleus, which contrasts the standard model of subcellular localization of RNAi complexes^1–3^. Furthermore, apart from our recent work^29–31,34,35^ and besides its evident prevalence in epithelia (Figs. 2–6 herein), junctional localization of RNAi complexes has been otherwise overlooked. In fact, our previously published findings using the canine MDCK cells show that this junctional localization may also be conserved across species^29,30^. The reason that this junctional association of RNAi complexes may have been missed is possibly due to the experimental models that have been traditionally used in the field, which include transformed cell lines, such as HeLa cells, or cells that don’t form stable, mature cell-cell junctions, such as fibroblasts, but not well-differentiated epithelial cells or tissues. Indeed, we have previously found that transformed colon cancer cells lack junctional localization of RNAi components^31^, whereas our findings presented here show that this is also absent from fibroblasts (Figs. 2–6). The use of highly proliferative transformed cells and fibroblasts in most studies has yielded abundant biochemical information that substantially moved the field forward. However, these cells lack the physiological context of normal, well-differentiated epithelial tissues, which comprise ~ 70% of the human body and are responsible for barrier formation. Epithelial tissues depend on adherens junction stability for their architecture and to form these barriers^32,33^. Since adherens junction formation requires cells to be in contact, we also particularly took care to grow cells in confluency, or to examine fully differentiated normal tissues that all exhibit fully formed, mature adherens junctions. It is also likely that previous studies didn’t take that factor into consideration, but examined cells in sub-confluent conditions, in this way missing the opportunity to identify junctional localization of RNAi components.

Two exceptions that we identified in the rule of RNAi junctional localization in well-differentiated epithelia are the HaCaT and MCF10A cells. HaCaT are well-differentiated immortalized skin keratinocytes that did form adherens junctions in our cultures, based on p120 staining (Figs. 1–5). However, p120 in these cells looks more diffused compared to that of Caco2 or ARPE-19 cells, denoting incomplete adherens junction maturation. Skin is a stratified epithelium and our stratified epithelial example, the esophageal tissue, exhibits junctional localization of PLEKHA7 and RNAi only at the apical-most layers of the tissue (Fig. 6). Therefore, it is likely that HaCaT cells may also have to be cultured in a stratified manner, to exhibit the same localization pattern of these complexes. Furthermore, although PLEKHA7, DROSHA and DGCR8 are recruited to adherens junctions of MCF10A cells, this is not the case for AGO2 and GW182, which fail to exhibit junctional localization in these cells. A possible explanation is that MCF10A cells, although immortalized and well-differentiated, lack a critical polarity component called Crumbs3 and don’t fully polarize in culture^41^. These observations may also provide hints for the mechanisms that are responsible for recruitment of RNAi components to the junctions. One of these may indeed be polarity complexes that are critical for adherens junction maturation, such as the Crumbs complex^42^. The other may be adherens junctions forming the structure called the zonula adherens. The zonula adherens forms in well-differentiated epithelia when adherens junctions tether to a tensile circumferential actomyosin ring^32,33^. Along these lines, we recently showed that recruitment and activity of AGO2 at adherens junctions depends on the presence of a structurally intact and tensile actomyosin cytoskeleton and on proper zonula adherens formation^34^. We also showed that it is not only PLEKHA7 that mediates this recruitment, but additional actin-binding proteins^34^, revealing a broader mode of regulation or RNAi at areas of cell-cell contact that depends on actomyosin mechanics rather than specific protein-protein interactions. Together, the above could explain why AGO2 and GW182 are not junctional in MCF10A cells, despite PLEKHA7 being present and junctional, or why no RNAi component is junctional in HaCaT cells, pointing towards a mechanism of junctional RNAi recruitment that depends not on a single protein component but on proper polarization and maturation of the epithelium and of the zonula adherens.

The above further underscore why assessing RNAi subcellular topology is critical to infer RNAi function and physiological role in each cell type, tissue, or condition. For example, localization of the microprocessor in paraspeckles enhances its pri-miRNA processing function through interaction with scaffolding long non-coding RNAs (lncRNAs)^12^, whereas its localization in the cytoplasm enables it as a cellular anti-viral defense mechanism^17–19^. Localization of AGO2 and of RISC in stress granules is critical for their silencing activity^14–16^, whereas their nuclear localization enables functions such transposon silencing, gene expression regulation, or regulation of stemness^22–24,26,27^. Differential localization of AGO2 has also been reported in pathological conditions, such as in the nucleus of colon cancer cells, in response to cell density^43^, or at the membrane of cancer cells and tumors^44,45^. We have shown that the adherens junctions – associated microprocessor and RISC complexes in polarized, well-differentiated cells engages and suppresses pro-tumorigenic mRNAs and acts to maintain epithelial cell homeostasis^29,31^. This is in line with our current findings demonstrating broad localization of these RNAi complexes at adherens junctions of epithelial cells and tissues, but not e.g. of fibroblasts or transformed cells, highlighting a potential role of the junctional RNAi machinery as an epithelial homeostatic mechanism. Moreover, our recent findings revealing regulation of the junctional AGO2 and of its miRNA-binding activity by the actin cytoskeleton, or its crosstalk with the extracellular matrix (ECM) align with similar findings of ECM – responsiveness and focal adhesion – association of AGO2 in endothelial cells and fibroblasts, point towards a RNAi machinery that is involved in the mechanosensitive regulation of the cell^34,35,46–48^.

In summary, our current study reveals RNAi localization patterns that is substantially broader compared to the standard model of RNAi localization. Among these patterns are extensive nuclear RISC and cytoplasmic microprocessor, as well as epithelial-specific junctional localization of both. These localization patterns are also supported by previous mechanistic studies by us and others and call for revisiting of the currently presented models in the literature. These findings can also open new avenues of investigation, especially regarding the physiological roles and interactions of the RNAi complexes. For example, there seems to be an extensive overlap of the microprocessor and RISC, either in the nucleus or in the cytoplasm, or at adherens junctions. This overlap has not been previously appreciated and begs the question on whether these RNA-binding complexes interact and to what extent. On the flipside, recruitment of the microprocessor and RISC to adherens junctions does not always exhibit the same pattern, such as in MCF10A cells (absence of junctional RISC), or in the tissues that we studied (more extensive presence of DROSHA at lateral junctions), implying for different modes of regulation of these complexes at adherens junctions. Altogether, these observations may spur future studies that will expand our understanding of the role of the RNAi machinery in cellular and tissue physiology.

## Methods

### Cell culture

In all comparisons, cells were used at strictly the same confluences and grown at 37°C, with 5% CO2. Cell lines were authenticated by the University of Arizona Genetics Core (via Science Exchange) and checked for misidentified, cross-contaminated, or genetically drifted cells. Cell lines tested negative for mycoplasma contamination (LookOut Mycoplasma PCR Detection Kit, Sigma-Aldrich). Human colon epithelial Caco2 cells (ATCC, cat# HTB-37) were grown in MEM cell culture medium (Corning, cat# MT10010CV), supplemented with 10% FBS (Gibco-Life Technologies, cat# A3160502), 1 mM sodium pyruvate (Gibco-Invitrogen, cat# 11360070-100 mM) and 1X non-essential amino-acid supplement (Gibco-Invitrogen, cat# 11140050-100x). HUVEC cells (Lonza, cat# CC-2517) were grown in endothelial cell growth medium (Sigma-Aldrich, cat# C-22010, plus supplement mix, cat# C-39216, with detach kit, cat# C-41200). MCF10A cells (ATCC, cat# CRL10317) were grown in DME/F12 (Fisher Scientific, cat# SH30023.01) with 5% horse serum (Invitrogen, cat# 16050122), 20 ng/ml EGF (Sigma-Aldrich, cat# E9644), 0.5 μg/ml hydrocortisone (Sigma-Aldrich, cat# H0888), 100 ng/ml Cholera Toxin (Sigma-Aldrich, cat# C8052), 10 μg/ml insulin (Sigma-Aldrich, cat# I18882)^49^. HaCaT cells^50^ were grown in McCoy’s (Fisher Scientific, cat# SH3020001) with 10% FBS. HepG2 (ATCC, cat# HB8065), were grown in MEM cell culture medium (Corning, cat# MT10010CV), supplemented with 10% FBS (Gibco-Life Technologies, cat# A3160502), 1 mM sodium pyruvate (Gibco-Invitrogen, cat# 11360070-100 mM), 1X non-essential amino-acids supplement (Gibco-Invitrogen, cat# 11140050-100x) with 2 mM L-Glutamine (Fisher Scientific, cat# MT25005CI). HeLa (ATCC, cat# CCL-2) were grown in DMEM with 10% heat-inactivated FBS. U2OS cells (ATCC, cat# HTB96) were grown in DMEM with 10% heat-inactivated FBS. HISM cells (ATCC, cat# CRL1692) were cultured in DMEM (Gibco - Fisher Scientific, cat# SH3002301) supplemented with 10% FBS. ARPE-19 cells (ATCC, cat# CRL-2302) were expanded in high glucose DMEM with pyruvate (Gibco - Fisher Scientific, cat# 11995073), with 10% FBS and 1% Antibiotic-Antimycotic (Gibco - Fisher Scientific, cat# 15240062) and grown on Transwell filters (Costar, 0.4 μm pore size; Corning, cat# 3460) to form polarized monolayers upon stepwise FBS removal^51^. Serum starvation (FBS free) conditions were instituted upon at least one media change before experiments were performed.

### Cell line immunofluorescence

All cell lines, except ARPE-19, were grown in 12 well plates on 18 mm sterile glass coverslips until they reached full confluence. ARPE-19 cells were grown on transwell inserts, as described above, stained on the inserts as described next, and then the membranes with the cells were mounted on coverslips for imaging. Cells were washed once with PBS and fixed with 100% methanol (Thermo Fisher Scientific) at −20°C for 7 min. Cells were then blocked with serum free Protein Block reagent (Dako) at RT for 1hr and stained with primary antibodies diluted in Antibody Diluent (Dako) overnight at 4°C. Cells were then washed three times with PBS, stained with the fluorescent-labeled secondary antibodies for 1 hr at RT, washed three times with PBS, co-stained with DAPI (Sigma-Aldrich), and mounted (Aqua-Poly/Mount; Polysciences). Images were acquired using Leica SP5 and SP8 confocal microscopes with 63x Plan-Apochromat 1.4NA DIC oil immersion objectives (Leica) and 405 nm, 488 nm, 594 nm, and 633 nm lasers. Image acquisition was done using Leica Application Suite Advanced Fluorescence X software at 1024×1024 resolution and with 0.5 μm intervals along the z-axis. Antibodies used were: PLEKHA7 (Sigma-Aldrich, cat# HPA038610), p120 (EMD Millipore, cat# 05-1567), DROSHA (Cell Signaling D29B1, cat#3364), DGCR8 (Sigma-Aldrich, cat# HPA019965), AGO2 (Abcam cat# AB156870), GW182 (Novus Biologicals Cat# NBP3-03014). Working dilutions: 1:50 – 1:500. Secondary antibodies used: Alexa 488 anti-mouse (Life Technologies, cat# A-11029), Alexa 488 anti-rabbit (Life Technologies, cat# A11034), Alexa 594 anti-mouse (Life Technologies, cat# A-11005), Alexa 594 anti-rabbit (Life Technologies, cat# A-11037), Alexa 647 anti- Rabbit (Life Technologies, cat# A21245) Alexa 647 anti- Mouse (Life Technologies cat# A21236). Working dilutions: 1:500.

### Tissue Multiplex immunofluorescence

Human tissues were obtained from the Hollings Cancer Center Biorepository, Medical University of South Carolina (MUSC). All research was performed in accordance with relevant guidelines and regulations, with the approval of the MUSC’s Institutional Review Board (IRB), under the protocol number Pro00062968. More specifically, under this protocol, the study was deemed as Not Human Research (NHR) by MUSC’s IRB, based on criteria set forth by the Code of Federal Regulations (45CFR46), since: a) the specimens and/or private information/data were not collected specifically for the currently proposed research project through and interaction/intervention with living individuals; and b) the investigator(s) including collaborators on the proposed research cannot readily ascertain the identity of the individual(s) to whom the coded private information or specimens pertain.

For multiplex immunofluorescence, slides were deparaffinized by immersing in xylene (Fisher Scientific, Hampton, NH) twice for 5 min each time. Samples were then rehydrated through a series of EtOH solutions (100-100-95-80-70-50%) and placed in distilled water. Deparaffinized slides were subjected to antigen retrieval for 32 mins at 95°C with EDTA and incubated with the respected antibodies for 32 mins at 37°C using a Ventana Discovery Ultra system (Roche). Antibodies used were: PLEKHA7 (Genetex, cat# GTX131146) at 1:200 dilution; DROSHA (Abnova, cat# PAB7156) at 1:100 dilution; AGO2 (ECM Biosciences, cat# AP5281) at 1:200 dilution; and E-cadherin (Cell Signaling Technologies; cat#3195) at 1:300 dilution. Slides were scanned using an Akoya Vectra Polaris scanner and images were analyzed using QuPath 0.2.0.

### Image Quantifications

To allow for comparisons, the same imaging parameters were used across conditions for all acquisitions. Images shown in figures are max projections of 3 single Z-slices to account for uneven cell thicknesses and ensure full representation of all junctional, cytoplasmic, and nuclear compartments. Line scans were performed using Fiji^52^ (National Institutes of Health), by drawing 12 μM lines spanning the nuclear and the cytoplasmic compartments and ending to the junctional compartment. We used DAPI staining as the nuclear reference and p120 staining as the junction-specific reference. Fluorescence intensity values were measured using the Plot Profile module in Fiji. For all measurements, sample size and related statistics are indicated in the respected figure legends. Statistics and graphs were all performed using Prism 10 (GraphPad).

### Immunoblotting

Whole cell extracts were obtained using RIPA buffer (50 mM Tris pH 7.4, BioRad cat# 1610719; 150 mM NaCl, Sigma-Aldrich cat# S9888-10K; 1% NP-40, Thermo Fisher Scientific cat# 507517565; 0.5% deoxycholic acid, Sigma-Aldrich cat#D6750-100G; and 0.1% SDS, Thermo Fisher Scientific, cat# BP166-500), supplemented with protease (Thermo Fisher Scientific, cat# 50550432) and phosphatase (Pierce Biotechnology, cat# P178420) inhibitors. Lysates were homogenized by passing through a 29-G needle and cleared by full-speed centrifugation for 5 min. Protein quantification was performed using a Pierce BCA Protein Assay (Pierce Biotechnology, cat# I23227). Protein extracts were mixed with Laemmli sample buffer at 2x final, separated by SDS-PAGE using 4–20% TGX gels (Bio-Rad, cat# 4568094), and transferred to 0.2 m nitrocellulose membranes (Bio-Rad, cat# 1704158) with the Bio-Rad^®^ Trans-Blot Turbo Transfer System. Membranes were blocked and blotted in 3% milk according to standard protocols. Antibodies used: PLEKHA7 (Sigma-Aldrich cat# HPA038610), p120 (EMD Millipore, cat# 05-1567), DROSHA, (Cell Signaling D29B1, cat#3364); DGCR8 (Abnova, cat# H00054487-M01), AGO2 (Abcam, cat# AB156870), GW182 (Santa Cruz, cat# sc-56314), Actin (Cell Signaling, cat# 4967). Antibodies were used at 1:500–1:1000 dilution. Secondary antibodies used: HRP-anti-mouse (Jackson ImmunoResearch, cat# 715-035-150), HRP-anti-rabbit (Jackson ImmunoResearch, cat# 711-035-152). Signals were detected by luminescence using Pierce ECL (Thermo Fisher, cat# 32209) using a Bio-Rad^®^ ChemiDoc Imaging System.

## Data Availability

Data is provided within the manuscript or supplementary information files.

## References

[1] Ha M. & Kim V. N. Regulation of microRNA biogenesis. Nat. Rev. Mol. Cell. Biol. 15, 509–524. 10.1038/nrm3838 (2014). 25027649

[2] Krol J., Loedige I. & Filipowicz W. The widespread regulation of microRNA biogenesis, function and decay. Nat. Rev. Genet. 11, 597–610. 10.1038/nrg2843 (2010). 20661255

[3] Gebert L. F. R. & MacRae I. J. Regulation of microRNA function in animals. Nat. Rev. Mol. Cell. Biol. 20, 21–37. 10.1038/s41580-018-0045-7 (2019). 30108335 PMC6546304

[4] Bartel D. P., Metazoan & MicroRNAs Cell 173, 20–51, doi:10.1016/j.cell.2018.03.006 (2018). 29570994 PMC6091663

[5] Fire A. Potent and specific genetic interference by double-stranded RNA in Caenorhabditis elegans. Nature 391, 806–811. 10.1038/35888 (1998). 9486653

[6] Liu J. Argonaute2 is the catalytic engine of mammalian RNAi. Sci. (New York N Y). 305, 1437–1441. 10.1126/science.1102513 (2004). 15284456

[7] Eulalio A., Huntzinger E. & Izaurralde E. GW182 interaction with Argonaute is essential for miRN-Amediated translational repression and mRNA decay. Nat. Struct. Mol. Biol. 15, 346–353. 10.1038/nsmb.1405 (2008). 18345015

[8] Lee Y. The nuclear RNase III Drosha initiates microRNA processing. Nature 425, 415–419. 10.1038/nature01957 (2003). 14508493

[9] Wu H., Xu H., Miraglia L. J. & Crooke S. T. Human RNase III is a 160-kDa protein involved in preribosomal RNA processing. J. Biol. Chem. 275, 36957–36965. 10.1074/jbc.M005494200 (2000). 10948199

[10] Billy E., Brondani V., Zhang H., Muller U. & Filipowicz W. Specific interference with gene expression induced by long, double-stranded RNA in mouse embryonal teratocarcinoma cell lines. Proc. Natl. Acad. Sci. U S A. 98, 14428–14433. 10.1073/pnas.261562698 (2001). 11724966 PMC64698

[11] Meister G. Human Argonaute2 mediates RNA cleavage targeted by miRNAs and siRNAs. Mol. Cell. 15, 185–197. 10.1016/j.molcel.2004.07.007 (2004). 15260970

[12] Jiang L. NEAT1 scaffolds RNA-binding proteins and the Microprocessor to globally enhance pri-miRNA processing. Nat. Struct. Mol. Biol. 24, 816–824. 10.1038/nsmb.3455 (2017). 28846091 PMC5766049

[13] Logan M. K., McLaurin D. M. & Hebert M. D. Synergistic interactions between Cajal bodies and the miRNA processing machinery. Mol. Biol. Cell. 31, 1561–1569. 10.1091/mbc.E20-02-0144 (2020). 32432989 PMC7521794

[14] Kedersha N., Ivanov P. & Anderson P. Stress granules and cell signaling: more than just a passing phase? Trends Biochem. Sci. 38, 494–506. 10.1016/j.tibs.2013.07.004 (2013). 24029419 PMC3832949

[15] Leung A. K., Calabrese J. M. & Sharp P. A. Quantitative analysis of Argonaute protein reveals microRNA-dependent localization to stress granules. Proc. Natl. Acad. Sci. U S A. 103, 18125–18130. 10.1073/pnas.0608845103 (2006). 17116888 PMC1838717

[16] Jakymiw A. Disruption of GW bodies impairs mammalian RNA interference. Nat. Cell. Biol. 7, 1267–1274. 10.1038/ncb1334 (2005). 16284622

[17] Wei X., Tang J., Lin C., Jiang X. & Review Non-canonical role of Drosha ribonuclease III. Int. J. Biol. Macromol. 253, 127202. 10.1016/j.ijbiomac.2023.127202 (2023). 37793530

[18] Shapiro J. S., Langlois R. A., Pham A. M. & Tenoever B. R. Evidence for a cytoplasmic microprocessor of pri-miRNAs. RNA 18, 1338–1346. 10.1261/rna.032268.112 (2012). 22635403 PMC3383965

[19] Aguado L. C. RNase III nucleases from diverse kingdoms serve as antiviral effectors. Nature 547, 114–117. 10.1038/nature22990 (2017). 28658212 PMC5846625

[20] Dai L. Cytoplasmic Drosha activity generated by alternative splicing. Nucleic Acids Res. 44, 10454–10466. 10.1093/nar/gkw668 (2016). 27471035 PMC5137420

[21] Link S., Grund S. E. & Diederichs S. Alternative splicing affects the subcellular localization of Drosha. Nucleic Acids Res. 44, 5330–5343. 10.1093/nar/gkw400 (2016). 27185895 PMC4914122

[22] Sala L. AGO2 silences mobile transposons in the nucleus of quiescent cells. Nat. Struct. Mol. Biol. 30, 1985–1995. 10.1038/s41594-023-01151-z (2023). 37985687

[23] Benhamed M., Herbig U., Ye T., Dejean A. & Bischof O. Senescence is an endogenous trigger for microRNA-directed transcriptional gene silencing in human cells. Nat. Cell. Biol. 14, 266–275. 10.1038/ncb2443 (2012). 22366686 PMC5423543

[24] Sarshad A. A. Argonaute-miRNA Complexes Silence Target mRNAs in the Nucleus of Mammalian Stem Cells. Mol Cell 71, 1040–1050 e1048, (2018). 10.1016/j.molcel.2018.07.020 30146314 PMC6690358

[25] Gagnon K. T., Li L., Chu Y., Janowski B. A. & Corey D. R. RNAi factors are present and active in human cell nuclei. Cell. Rep. 6, 211–221. 10.1016/j.celrep.2013.12.013 (2014). 24388755 PMC3916906

[26] Griffin K. N. Widespread association of the Argonaute protein AGO2 with meiotic chromatin suggests a distinct nuclear function in mammalian male reproduction. Genome Res. 32, 1655–1668. 10.1101/gr.276578.122 (2022). 36109149 PMC9528986

[27] Ahlenstiel C. L. Direct evidence of nuclear Argonaute distribution during transcriptional silencing links the actin cytoskeleton to nuclear RNAi machinery in human cells. Nucleic Acids Res. 40, 1579–1595. 10.1093/nar/gkr891 (2012). 22064859 PMC3287199

[28] Robb G. B., Brown K. M., Khurana J. & Rana T. M. Specific and potent RNAi in the nucleus of human cells. Nat. Struct. Mol. Biol. 12, 133–137. 10.1038/nsmb886 (2005). 15643423

[29] Kourtidis A. Distinct E-cadherin-based complexes regulate cell behaviour through miRNA processing or Src and p120 catenin activity. Nat. Cell. Biol. 17, 1145–1157. 10.1038/ncb3227 (2015). 26302406 PMC4975377

[30] Kourtidis A. Cadherin complexes recruit mRNAs and RISC to regulate epithelial cell signaling. J. Cell. Biol. 216, 3073–3085. 10.1083/jcb.201612125 (2017). 28877994 PMC5626537

[31] Nair-Menon J. Predominant Distribution of the RNAi Machinery at Apical Adherens Junctions in Colonic Epithelia Is Disrupted in Cancer. Int. J. Mol. Sci. 21 10.3390/ijms21072559 (2020). PMC7177752 32272708

[32] Takeichi M. Dynamic contacts: rearranging adherens junctions to drive epithelial remodelling. Nat. Rev. Mol. Cell Biol. 15, 397–410. 10.1038/nrm3802 (2014). 24824068

[33] Harris T. J. & Tepass U. Adherens junctions: from molecules to morphogenesis. Nat. Rev. Mol. Cell. Biol. 11, 502–514. 10.1038/nrm2927 (2010). 20571587

[34] Bridges M. C. Actin-dependent recruitment of AGO2 to the zonula adherens. Molecular Biology of the Cell 34, ar129, (2023). 10.1091/mbc.E22-03-0099-T PMC10848941 37819702

[35] Daulagala A. C. & Kourtidis A. E. C. M. Substrates Impact RNAi Localization at Adherens Junctions of Colon Epithelial Cells. Cells 11, 3740. 10.3390/cells11233740 (2022). 36497003 PMC9737857

[36] Grasset E., Pinto M., Dussaulx E., Zweibaum A. & Desjeux J. F. Epithelial properties of human colonic carcinoma cell line Caco-2: electrical parameters. Am. J. Physiol. 247, C260–267. 10.1152/ajpcell.1984.247.3.C260 (1984). 6476109

[37] Hidalgo I. J., Raub T. J. & Borchardt R. T. Characterization of the human colon carcinoma cell line (Caco-2) as a model system for intestinal epithelial permeability. Gastroenterology 96, 736–749 (1989). 2914637

[38] Sambuy Y. The Caco-2 cell line as a model of the intestinal barrier: influence of cell and culture-related factors on Caco-2 cell functional characteristics. Cell. Biol. Toxicol. 21, 1–26. 10.1007/s10565-005-0085-6 (2005). 15868485

[39] Kourtidis A., Ngok S. P. & Anastasiadis P. Z. p120 catenin: an essential regulator of cadherin stability, adhesion-induced signaling, and cancer progression. Prog Mol. Biol. Transl Sci. 116, 409–432. 10.1016/B978-0-12-394311-8.00018-2 (2013). 23481205 PMC4960658

[40] Sharma N. R. Cell Type- and Tissue Context-dependent Nuclear Distribution of Human Ago2. J. Biol. Chem. 291, 2302–2309. 10.1074/jbc.C115.695049 (2016). 26699195 PMC4732213

[41] Fogg V. C., Liu C. J. & Margolis B. Multiple regions of Crumbs3 are required for tight junction formation in MCF10A cells. J. Cell. Sci. 118, 2859–2869. 10.1242/jcs.02412 (2005). 15976445

[42] Buckley C. E. St Johnston, D. Apical-basal polarity and the control of epithelial form and function. Nat. Rev. Mol. Cell. Biol. 23, 559–577. 10.1038/s41580-022-00465-y (2022). 35440694

[43] Johnson K. C. Nuclear localization of Argonaute 2 is affected by cell density and may relieve repression by microRNAs. Nucleic Acids Res. 52, 1930–1952. 10.1093/nar/gkad1155 (2024). 38109320 PMC10899759

[44] Shankar S. An essential role for Argonaute 2 in EGFR-KRAS signaling in pancreatic cancer development. Nat. Commun. 11, 2817. 10.1038/s41467-020-16309-2 (2020). 32499547 PMC7272436

[45] Lin M. C. Ago2/CAV1 interaction potentiates metastasis via controlling Ago2 localization and miRNA action. EMBO Rep. 25, 2441–2478. 10.1038/s44319-024-00132-7 (2024). 38649663 PMC11094075

[46] Daulagala A. C. The epithelial adherens junction component PLEKHA7 regulates ECM remodeling and cell behavior through miRNA-mediated regulation of MMP1 and LOX. bioRxiv, (2024). 10.1101/2024.05.28.596237

[47] Moro A. MicroRNA-dependent regulation of biomechanical genes establishes tissue stiffness homeostasis. Nat. Cell Biol. 21, 348–358. 10.1038/s41556-019-0272-y (2019). 30742093 PMC6528464

[48] Kumar A., Tanaka K. & Schwartz M. A. Focal adhesion-derived liquid-liquid phase separations regulate mRNA translation. bioRxiv 10.1101/2023.11.22.568289 (2024). PMC12201949 40568958

[49] Debnath J., Muthuswamy S. K. & Brugge J. S. Morphogenesis and oncogenesis of MCF-10A mammary epithelial acini grown in three-dimensional basement membrane cultures. Methods 30, 256–268 (2003). 12798140 10.1016/s1046-2023(03)00032-x

[50] Talwar S. Overexpression of RNA-binding protein CELF1 prevents apoptosis and destabilizes pro-apoptotic mRNAs in oral cancer cells. RNA Biol. 10, 277–286. 10.4161/rna.23315 (2013). 23324604 PMC3594286

[51] Thurman J. M. Oxidative stress renders retinal pigment epithelial cells susceptible to complement-mediated injury. J. Biol. Chem. 284, 16939–16947. 10.1074/jbc.M808166200 (2009). 19386604 PMC2719331

[52] Schindelin J. Fiji: an open-source platform for biological-image analysis. Nat. Methods. 9, 676–682. 10.1038/nmeth.2019 (2012). 22743772 PMC3855844

